# Lessons for the clinical nephrologist: acute kidney injury during therapy with apixaban

**DOI:** 10.1007/s40620-023-01781-y

**Published:** 2023-12-06

**Authors:** Gisella Vischini, Serena Speciale, Agnieszka Przygocka, Laura Martano, Gaetano La Manna, Olga Baraldi

**Affiliations:** 1 Nephrology, Dialysis and Kidney Transplant Unit, IRCCS Azienda Ospedaliero-Universitaria di Bologna, Bologna, Italy; 2 Department of Medical and Surgical Sciences (DIMEC), Alma Mater Studiorum-University of Bologna, Via Giuseppe Massarenti 9, Bologna, Italy

**Keywords:** Anticoagulant-related nephropathy, Acute kidney injury, Chronic kidney disease, Leucocytoclastic vasculitis, Apixaban

## The Case

An 81-year-old Caucasian male was hospitalized with respiratory distress due to pneumonia with pleural effusion. His medical history was remarkable for chronic kidney disease with serum creatinine (sCreat) of 1.6 mg/dL, chronic obstructive pulmonary disease, and atrial fibrillation on therapy with apixaban. Laboratory tests showed worsening of renal function during the infection (serum creatinine up to 2.2 mg/dL). He was treated with antibiotic therapy and pleural drainage; during hospitalization, anticoagulant therapy was modified to enoxaparin. Given the improvement of the patient’s general conditions, apixaban was resumed at hospital discharge.

Ten days later a chest computed tomography was performed as follow up and documented new ground glass areas and pulmonary consolidations, therefore the patient was referred to the emergency department. Physical examination showed pitting edema and bilateral rhonchi. Blood analysis revealed severe renal dysfunction (sCreat, 10.78 mg/dL; serum urea, 264 mg/dL) and hematuria. The patient was oliguric (urine output of 300 mL/day). Apixaban therapy was suspended. Since vasculitis was suspected, steroid therapy was started (methylprednisolone 125 mg/day with subsequent tapering). Given the persistence of oliguria hemodialysis was initiated.

Antinuclear antibodies, antineutrophil cytoplasmic antibodies and anti-glomerular basement membrane antibodies tested negative. Further workup ruled out hepatitis B and C as well as tuberculosis infection. Urine sediment analysis documented dysmorphic red blood cell casts. He also presented petechial eruption on his hands and lower limbs, therefore a skin biopsy was carried out and showed leukocytoclastic vasculitis. Fibrobronchoscopy with bronchoalveolar lavage was performed revealing evidence of alveolar hemorrhage.

In order to better define the etiology of persistent kidney injury (as indicated by [1] in case of unexplained worsening of renal function in a patient treated with anticoagulants), to exclude drug-induced vasculitis and to evaluate the need for further immunosuppressive therapy, the patient underwent ultrasound-guided kidney biopsy. Subsequently, on the same day, he presented hypotension (systolic blood pressure of 60 mmHg) and acute anemia (hemoglobin level drop from 11.1 to 8.7 g/dL). Urgent computed tomography scan showed hemorrhage due to left renal artery damage, thus artery embolization was performed, with temporary stabilization of the patient’s conditions. However, in the following days he presented hemodynamic instability requiring inotropic therapy, with poor tolerance to hemodialysis. Multiple organ failure ensued and the patient died on the 25th day of hospitalization.

Kidney biopsy showed acute tubular damage and glomerular hemorrhage with erythrocyte casts in Bowman’s space and renal tubules, compatible with the diagnosis of anticoagulant-related nephropathy. The glomeruli were unremarkable: no intra- or extracapillary proliferation was found, therefore vasculitis was excluded. Immunofluorescence was negative for immune complex deposits.

## Lessons for the clinical nephrologist

Anticoagulant-related nephropathy is a newly recognized cause of acute kidney injury, and the mechanism of kidney damage is still not completely understood. It is characterized by glomerular hemorrhage and by the presence of erythrocyte casts in renal tubules. Glomeruli are normal or present only minor changes and anticoagulant-related nephropathy should be suspected in case of disproportion between a high number of red blood cell casts, signs of acute tubular necrosis and minimal or absent glomerular lesions [2, 3]. Initially, it was reported in patients receiving warfarin, but subsequently the association with direct oral anticoagulant therapy was also documented [2]. The postulated mechanism is tubular obstruction by erythrocyte casts with release of free hemoglobin from red blood cells leading to increased oxidative stress [3]. The available data suggest that injury is not due to coagulopathy alone but that other factors causing pre-existing glomerular damage must be present [3]. The main risk factors are chronic kidney disease, age, diabetes mellitus, hypertension and heart failure [1, 2].

Data on direct oral anticoagulant use and its safety in chronic kidney disease are limited. A recent review of available studies which included patients with chronic kidney disease and atrial fibrillation who were on therapy with direct oral anticoagulants compared to warfarin revealed a trend towards less major bleeding with lower thromboembolic risk in the novel anticoagulant group [4]. Furthermore, several trials (including post hoc analysis) demonstrated lower risk of acute kidney injury and slower chronic kidney disease progression with novel agents compared to warfarin [4].

Nevertheless, cases of anticoagulant-related nephropathy in patients on therapy with direct oral anticoagulants were reported [2]. This emphasizes the need for particular attention to, and monitoring of, patients with chronic kidney disease on anticoagulant treatment, both with older and novel agents. Furthermore, a recent analysis of anticoagulant-related nephropathy showed that its prevalence is also not uncommon in patients with previously reported normal kidney function (although warfarin is associated with higher risk than direct oral anticoagulant therapy) [1, 5]. Therefore, this recently recognized cause of acute kidney injury should be considered in cases of otherwise unexplainable worsening of renal function in patients on anticoagulant therapy. Renal biopsy is seldom performed because of the high risk of complications, but can provide valuable information. There are reports in the literature where anticoagulant-related nephropathy was the first clinical presentation of underlying glomerular disease (such as IgA nephropathy) [6] which further supports the usefulness of histological examination in this context.

Currently there are no guidelines addressing the management of anticoagulant-related nephropathy. General recommendations suggest optimization of anticoagulant therapy to a therapeutic range, moreover, on the basis of available data, switching to direct oral anticoagulants in patients on warfarin and reducing the dose in patients already receiving novel agents has been suggested [1, 7]. Temporary discontinuation of anticoagulant therapy, when possible, is also proposed. Some authors suggest a course of corticosteroid treatment, with careful evaluation of the risk of complications, in particular life-threatening infections [7]. Prognosis is uncertain and the mortality rate is high; many patients do not recover kidney function [1, 2].

**Fig. 1:**
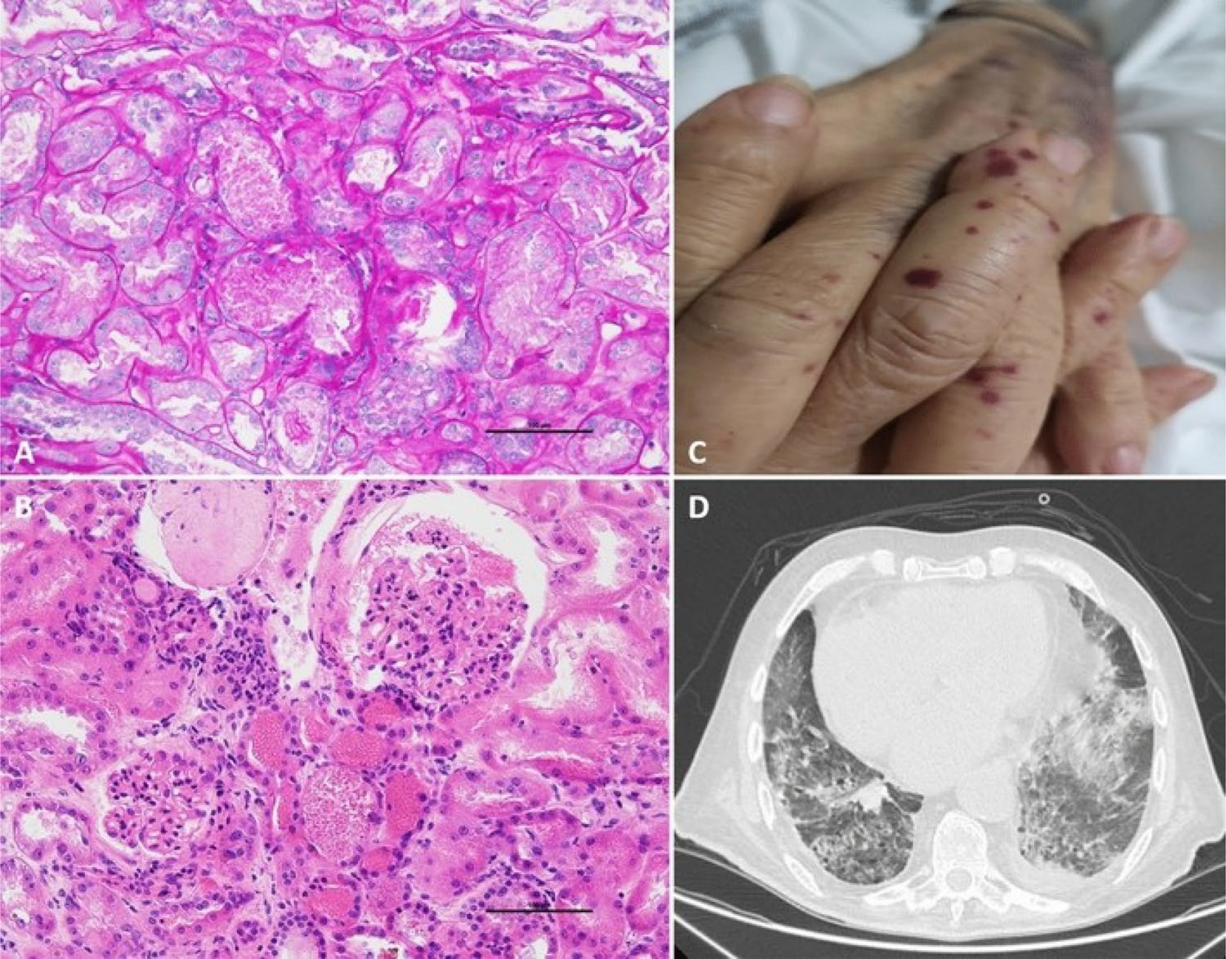
**A** Light microscopy of the cortical renal parenchyma, periodic acid-Schiff staining shows acute tubular injury with loss of brush border and simplification of tubular cells with presence of red blood cell casts (PAS ×20; scale bar 100 μm). **B** Hematoxylin and eosin staining demonstrates erythrocyte casts in tubular lumen and in Bowman’s space (H&E ×20; scale bar 100 μm). **C** Petechiae which appeared in course of acute kidney injury during the hospitalization. **D** High resolution computed tomography shows extensive ground glass areas and signs of alveolar hemorrhage

In the literature there are reports of anticoagulant-related nephropathy with leukocytoclastic vasculitis caused by warfarin [8] and direct oral anticoagulant-related cutaneous vasculitis without kidney injury [9]. Our patient presented biopsy-proven anticoagulant nephropathy and the vasculitic cutaneous lesions were most likely induced by the same drug. This case reminds us that acute kidney injury with clinical presentation suggestive of systemic vasculitis can be a manifestation of anticoagulant-related nephropathy (Fig. 1).

### Learning points

- Anticoagulant-related nephropathy is a recently recognized and most likely underestimated cause of acute renal injury.
- It is associated with both older and novel anticoagulant agents, although available data demonstrate higher risk on therapy with warfarin than with direct oral anticoagulants.
- Preexisting chronic kidney disease is one of the main risk factors, therefore careful follow up is necessary in this group of patients on anticoagulant therapy.
- Kidney biopsy provides valuable information about the kidney injury but is not always performed due to the increased risk of complications.
- There are no guidelines regarding treatment of anticoagulant-related nephropathy but a switch to direct oral anticoagulants in patients on warfarin, dose reduction in patients already on therapy with novel agents, temporary discontinuation of anticoagulant therapy and a course of corticosteroid treatment may be considered.

### Supplementary Information

Below is the link to the electronic supplementary material.

Supplementary file1 (DOCX 16 kb)
