# Supplementary material for: Lessons for the clinical nephrologist: acute kidney injury during therapy with apixaban
Source: J Nephrol. 2023 Dec 6;37(2):511–3. doi: 10.1007/s40620-023-01781-y (PMC11043096; doi:10.1007/s40620-023-01781-y)
Supplement: Supplementary file 1 — Supplementary file1 (DOCX 16 kb) [file 40620_2023_1781_MOESM1_ESM.docx]

**Supplementary material**

1. Brodsky SV, Mhaskar NS, Thiruveedi S, Dhingra R, Reuben SC, Calomeni E, Ivanov I, Satoskar A, Hemminger J, Nadasdy G, Hebert L, Rovin B, Nadasdy T. Acute kidney injury aggravated by treatment initiation with apixaban: Another twist of anticoagulant-related nephropathy. Kidney Res Clin Pract. 2017 Dec; 36(4):387-392.
2. Ware K, Qamri Z, Ozcan A, Satoskar AA, Nadasdy G, Rovin BH, Hebert LA, Nadasdy T, Brodsky SV: N-acetylcysteine ameliorates acute kidney injury but not glomerular hemorrhage in an animal model of warfarin-related nephropathy. Am J Physiol Renal Physiol 2013; 304:F1421–F1427.
3. Brodsky SV, Nadasdy T, Rovin BH et al. Warfarin-related nephropathy occurs in patients with and without chronic kidney disease and is associated with an increased mortality rate. Kidney Int 2011; 80: 181–189.
4. Kimachi M, Furukawa TA, Kimachi K, et al. Direct oral anticoagulants versus warfarin for preventing stroke and systemic embolic events among atrial fibrillation patients with chronic kidney disease. Cochrane Database Syst Rev 2017; 11: CD011373.
5. Yao X, Tangri N, Gersh BJ, et al. Renal Outcomes in Anticoagulated Patients With Atrial Fibrillation. J Am Coll Cardiol 2017; 70: 2621–2632.
6. Bohm M, Ezekowitz MD, Connolly SJ, et al. Changes in Renal Function in Patients With Atrial Fibrillation: An Analysis From the RE-LY Trial. J Am Coll Cardiol 2015; 65: 2481–2493.
7. Fordyce CB, Hellkamp AS, Lokhnygina Y, et al. On-Treatment Outcomes in Patients With Worsening Renal Function With Rivaroxaban Compared With Warfarin: Insights From ROCKET AF. Circulation 2016; 134: 37–47.
